# Maternal smoking and the retinoid pathway in the developing lung

**DOI:** 10.1186/1465-9921-13-42

**Published:** 2012-06-01

**Authors:** Sara E Manoli, Lacey A Smith, Carrie A Vyhlidal, Chang Hyeok An, Yolanda Porrata, Wellington V Cardoso, Rebecca M Baron, Kathleen J Haley

**Affiliations:** 1 Department of Medicine, Division of Pulmonary and Critical Care, Brigham and Women’s Hospital, Boston, MA, USA; 2 Pulmonary Center of Boston University School of Medicine, Boston, MA, USA; 3 Division of Pediatric Clinical Pharmacology, Children’s Mercy Hospital and Clinics, Kansas City, MO, USA

**Keywords:** Maternal smoking, Lung development, Retinoic acid

## Abstract

**Background:**

Maternal smoking is a risk factor for pediatric lung disease, including asthma. Animal models suggest that maternal smoking causes defective alveolarization in the offspring. Retinoic acid signaling modulates both lung development and postnatal immune function. Thus, abnormalities in this pathway could mediate maternal smoking effects. We tested whether maternal smoking disrupts retinoic acid pathway expression and functioning in a murine model.

**Methods:**

Female C57Bl/6 mice with/without mainstream cigarette smoke exposure (3 research cigarettes a day, 5 days a week) were mated to nonsmoking males. Cigarette smoke exposure continued throughout the pregnancy and after parturition. Lung tissue from the offspring was examined by mean linear intercept analysis and by quantitative PCR. Cell culture experiments using the type II cell-like cell line, A549, tested whether lipid-soluble cigarette smoke components affected binding and activation of retinoic acid response elements *in vitro*.

**Results:**

Compared to tobacco-naïve mice, juvenile mice with tobacco toxin exposure had significantly (P < 0.05) increased mean linear intercepts, consistent with an alveolarization defect. Tobacco toxin exposure significantly (P < 0.05) decreased mRNA and protein expression of retinoic acid signaling pathway elements, including retinoic acid receptor alpha and retinoic acid receptor beta, with the greatest number of changes observed between postnatal days 3–5. Lipid-soluble cigarette smoke components significantly (P < 0.05) decreased retinoic acid-induced binding and activation of the retinoic acid receptor response element in A549 cells.

**Conclusions:**

A murine model of maternal cigarette smoking causes abnormal alveolarization in association with altered retinoic acid pathway element expression in the offspring. An *in vitro* cell culture model shows that lipid-soluble components of cigarette smoke decrease retinoic acid response element activation. It is feasible that disruption of retinoic acid signaling contributes to the pediatric lung dysfunction caused by maternal smoking.

## Introduction

Maternal smoking is an important risk factor for pediatric lung dysfunction, including asthma [1-6]. Children of mothers who smoke cigarettes have increased risk of lower respiratory tract infections and wheezing during their first year, and higher incidence of persistent wheeze and doctor-diagnosed asthma [4,5,7,8]. Additionally, maternal smoking is associated with increased frequency of asthma exacerbations among children [5,9]. Nonsmoking adults who report a history of childhood tobacco smoke exposure have lower baseline FEV-1 and a more rapid decline in pulmonary function tests after starting cigarette smoking [10,11]. Therefore, environmental insults such as tobacco toxin exposure during lung development may have a life-long impact on lung growth and function. The possibility of an early life insult leading to increased vulnerability to postnatal disease has been termed the “fetal origins” or “developmental origins” hypothesis [12,13].

Given that approximately 20% women between ages 18 – 44 years report current smoking, and current maternal smoking is reported in approximately 1 in 7 pregnancies in the United States [14,15], effective approaches to the problem of tobacco smoke-associated pediatric lung dysfunction will combine anti-smoking education with mechanistically-based therapies. Currently, however, the mechanisms of lung injury in children exposed to maternal smoking have not been fully clarified.

One possible pathway that could mediate some of the lung abnormalities caused by maternal smoking is the retinoic acid (RA) signaling pathway. RA is produced by vitamin A metabolism by successive oxidative reactions from dietary precursors including retinyl esters and carotenoids such as beta carotene [16,17]. The retinoids are well-described as critical mediators of alveolar development [18-22] and are also involved in the maintenance of normal postnatal lung epithelium and immune responses [18,23-25]. Cigarette smoking causes abnormalities in retinol levels in rodents [26]. In humans, cigarette smokers have decreased beta carotene compared to nonsmokers [27,28]; this decrease exceeds that explained by differences in dietary intake [29]. The data regarding changes in serum retinol levels in cigarette smokers are less consistent. Several studies comparing serum retinol levels in smokers versus nonsmokers have not demonstrated statistically significant differences [30-33]. In contrast, a large study (over 600 subjects in each group) did demonstrate lower mean serum retinol levels in current smokers compared to former smokers [28].

We hypothesized that prenatal exposure to tobacco toxins would decrease elements of the retinoic acid signaling pathway in the immature lung. We tested this hypothesis by examining lung tissue from mice with exposure to cigarette smoke byproducts during development and by examining an *in vitro* cell culture model to determine if lipid-soluble cigarette smoke extract caused abnormal RA receptor activation. Our findings indicate that maternal cigarette smoke decreases expression of several retinoic acid pathway components in the lungs of the offspring, and that these effects persist well into the postnatal period.

## Methods

All of the animal protocols used in this study were reviewed and approved in advance by the Harvard Medical School institutional review board (IACUC).

### Animal model

Study animals were housed in pathogen-free conditions, with ad libitum food and water in microisolator cages. Beginning at 10 weeks of age, female C57Bl/6 mice (Charles River Laboratories, Waltham, MA) received daily exposure to the smoke of 3 3R4F research cigarettes (University of Kentucky), 5 times per week. Control mice were exposed to filtered air using a dedicated HEPA Tecniplast SLIMLine™ filtration system (Tecniplast, Westchester, PA). The cigarette smoke was delivered using a custom-built apparatus that provides mainstream cigarette smoke exposure to the nose and face of the mouse. The exposures were initiated gradually, with smoke from 1 cigarette on the first day, 2 on the second day, and 3 daily thereafter. The cigarette smoke was delivered from sequential cigarettes during one session lasting approximately 30 minutes per mouse. The mice were continuously observed during the smoke exposure, and promptly removed from the smoke exposure chamber if any signs of distress were noted. After a 2-wk acclimation period, the females were mated to nonsmoking males. Cigarette smoke exposures were continued throughout pregnancy and after parturition. The pups were randomly allocated among the experimental groups. Tissue samples from the pups were collected after euthanasia following recommended IACUC prototols: carbon dioxide followed by excision of vital organs. At time points up to P10, tracheal transaction/decapitation was also performed after establishing carbon dioxide-induced narcosis by approximately 5 minutes of carbon dioxide exposure. The presence of narcosis was verified by observing the absence of breathing movements and the lack of response to tail pinch. Lung tissue samples from the offspring were collected on the day of birth (P0), postnatal day 3 (P3), P5, P7, P10, and P14 for RNA and protein analyses. These time points were chosen to sample the early neonatal period through the time of rapid alveolarization. Due to size constraints, whole lung samples were collected from P0 mice. Lung tissue samples from subsequent time points were obtained from areas located at least 3 – 5 mm distal to the lung hilum. This sampling strategy permitted examination of the distal lung areas, where alveolarization occurs. Both RNA and protein analyses included a total of at least 4 pups from the litters of at least 2 females. Additional P14 mice with/without tobacco toxin exposure were analyzed for changes in lung histology, as outlined below in “Histological Analyses”. Placenta and serum samples were harvested from additional pregnant females with (N = 8) and without (N = 8) cigarette smoke exposure at embryonic day e15.5 and e17.5.

The total particulates from the cigarette smoking chambers were measured by connecting the smoke chamber to a dry gas meter (catalog # GNM G1.6 T, AEM, Romania) in-line with an air monitoring filter holder. A 12 mm diameter borosilicate air monitoring filter (catalog # TX40HI20WW, Pallflex® Emfab™ filter, Pall Life Sciences) was weighed, and placed into the filter holder, and the smoking machine activated with a 3R4F cigarette After 8 minutes of exposure, the paper was weighed, and the total particulates calculated as follows: Total particulates = change in filter weight/ change in dry gas volume. Measurement was repeated, and an average total particulate measure calculated.

### Histological analysis

Changes in airspace architecture were estimated by calculating the mean linear intercept, which is an estimate of the average difference between gas exchange surfaces [34]. For these studies, lung tissue from P14 mice with (n = 16) and without (n = 12) exposure to tobacco toxins during development was inflated to 25 cm water pressure, and fixed in 10% formalin (Thermo Fisher Scientific Inc., Waltham, MA). The lungs were routinely processed and infiltrated with paraffin, sectioned in the mid-sagittal line prior to embedding in paraffin with the midsagittal line oriented to the block face, routinely sectioned into 5-microns thick sections, and stained with hematoxylin and eosin (Sigma -Aldrich®, St. Louis, MO). Using random numbers to determine sampling areas, five independent, uniform random 20X fields that were located within 100 microns of the pleural surface were digitally photographed using a Leica DMLB microscope interfaced with a Leica DFC480 digital camera (Leica Microsystems, Inc., Buffalo Grove, IL) by a blinded experienced reader (KH). Vascular structures and bronchi were identified by the typical appearance of the endothelial and epithelial cells, respectively, and digitally isolated from the areas to be measured, so that only distal airspaces were analyzed. The images were analyzed using Image J image analysis software (http://rsb.info.nih.gov/ij/) to determine the mean linear intercept as a measure of the average distance between gas exchange surfaces, as outlined in the American Thoracic Society/European Respiratory Society Standards for Quantitative Assessment of Lung Structure [34].

### Cotinine measurements

Crude placenta tissue extracts were prepared from 100–150 mg mouse placenta tissue by homogenization in 200 μl homogenization buffer (50 mM Tris–HCl, 150 mM KCl, 2 mM EDTA, pH. 7.5). Homogenates were centrifuged at 1000 X G for 15 minutes at 4°C. Cotinine was detected using 10 μl placenta extract or blood (diluted 1:2 in tissue homogenization buffer) with the Cotinine Direct ELISA kit (Calbiotech, Spring Valley, CA) according to manufacturer’s directions. Standard curves were prepared from serial dilutions of cotinine from 100 ng/ml to 1 ng/ml. Cotinine concentrations in placenta extracts and blood were calculated from a 4-parameter Logistic/Log analysis of the standard curves (SigmaPlot 6.0) as previously described [35].

### Real time (quantitative) PCR

RNA extraction was performed using TriReagent (Molecular Research Center, Inc.), according to manufacturer’s instructions, and was followed by DNase I (Invitrogen/Life Technologies, Grand Island, NY) treatment and reverse transcription to cDNA (Retroscript®, Ambion/Life Technologies) as previously described [36]. Real time polymerase chain reaction (qPCR) using validated Taqman primers and probes (Applied Biosystems/Life Technologies) was performed as previously described [36]. The catalog numbers for the primer/probe sets are given in Table 1, below. All of the primers except for 18 S spanned an intron.

**Table 1:** Catalog numbers for qPCR primer/probe sets

**Primer/Probe Set**	**Catalog Number**
Retinaldehyde dehydrogenase-1	Mm 00657317_m1
Retinaldehyde dehydrogenase-2	Mm00501306_m1
RA receptor alpha	Mm 00436264 –m1
RA receptor beta	Mm 01319680_m1
RA receptor gamma	Mm 00441083_m1
Retinoid X receptor alpha	Mm 01332431_m1
Retinoid X receptor beta	Mm 00441193_m1
Retinoid X receptor gamma	Mm 00436410_m1
Nuclear receptor subfamily 2, group F, member 2	Mm00772789_m1
Cytochrome P450 26b1	Mm00558507_m1
Vascular Endothelial Growth Factor-α	Mm00437304_m1
Transforming Growth Factor-beta	Mm03024053_m1
Surfactant apoprotein A	Mm00499170_m1
Surfactant apoprotein B	Mm00455681_m1
18 S (eukaryotic)	Hs 99999901_s1

### Protein extraction and western blotting analyses

Lung tissue harvested as outlined above from mice at ages postnatal day 3, 5, and 7 was snap frozen for protein extraction. The used of distal lung tissue enriched the samples for areas of developing alveoli. Total protein was extracted using Bio-Rad Protein Extraction kit (Bio-Rad Life Science Research, Hercules, CA) followed by Bio-Rad 2D cleanup kit (Bio-Rad), following manufacturer’s instructions, and then quantitated using bicinchoninic acid assay (Sigma). The protein samples were then analyzed by Western blotting, which was performed with modified method as previously described [37]. Briefly, after blocking for 1 h at room temperature with 5% nonfat dry milk in TBST buffer, the membranes were incubated with primary antibodies overnight at 4°C. Rabbit anti-RXRalpha (sc553, used at 200 ng/ml), RARalpha (sc551, used at 200 ng/ml), RARbeta (sc552, used at 200 ng/ml) and mouse anti-RARgamma (sc7387, used at 200 ng/ml) antibodies were obtained from Santa Cruz Biotechnology, Inc., Santa Cruz, CA. Mouse anti-beta actin (A2228, clone AC-74, used at 400 ng/ml) was purchased from Sigma. After brief washing, the membranes were incubated with horseradish peroxidase-conjugated secondary antibodies for 15 min at room temperature, extensively washed in TBST buffer, and then bands of the anticipated molecular weights were detected with chemiluminescence (Amersham ECL Plus™ Western Detection Kit, GE Healthcare Biosciences, Pittsburgh, PA). The blots were visualized by ChemiDoc XRS + System (Bio-Rad), and densitometry was performed to compare the relative intensities of the bands.

### Cell culture and transient transfection

A549 cells (ATCC) were maintained in Dulbelcco’s Modified Eagle Medium with 10% fetal bovine serum (Invitrogen Life Sciences), 1% L-glutamine (GIBCO/Invitrogen Life Sciences), and 2% penicillin/streptomycin (10,000 units penicillin/ml and 10 mg streptomycin/ml, Sigma). Transfection experiments were performed using standard protocols [38]. The transfection reagent was Fugene 6 (Roche Applied Science, Indianapolis, IN), used following the manufacturer’s instructions, with a 3:1 ratio of Fugene to total plasmid DNA. The plasmids used were a luciferase reporter plasmid containing RA response element (Panomics/Affymetrix Inc., Santa Clara, CA), empty vector (negative control plasmid, Panomics), pGL2 (Promega, Madison, WI; positive control plasmid, generous gift of M. Layne, Ph.D.), and a beta-galactosidase expression plasmid (used for normalizing luciferase activity, generous gift of M. Layne). Approximately 1.5 hours after transfection, cultures were treated with cigarette smoke condensate (CSC, Murty Pharmaceuticals Incorporated, Lexington, KY) at concentrations of 0.25% and 0.50% total volume and 1 μM all-trans retinoic acid (Sigma). Cultures were incubated overnight, and at approximately 14 h a second application of CSC was added to the medium, at 0.25% and 0.50% concentration. Four hours after the second dose of CSC, luciferase activity was examined after luciferase substrate (Promega) was added to cell lysates. Transfection efficiency was assessed by beta galactosidase activity, following standard protocols [39]. Cell toxicity was evaluated by lactate dehydrogenase release using the *In Vitro* Toxicology Assay Kit, Lactate Dehydrogenase based (TOX7, Sigma), following the manufacturer’s instructions.

### Electrophoretic mobility shift assays

A549 cells were split into 10 cm cell culture plates, allowed to grow for 24 h (to approximately 80% confluency), and then treated overnight with 1 μM retinoic acid and either 0.5% CSC or 0.5% dimethyl sulfoxide (diluent, DMSO, Sigma). Cultures were treated with a second dose of CSC or DMSO at 14 h after the initial dose. Nuclear extracts were harvested at 15 min, 30 min, 60 min, and 120 min after the second dose of CSC or DMSO using standard protocols [40], and nuclear protein was quantified using the Bradford Assay (Bio-Rad). Electrophoretic mobility shift assays (EMSA) were performed using standard protocols [41], using 10 μg of nuclear protein with a [γ-^32^P] ATP radiolabeled double-stranded DNA probe to detect a consensus sequence for the most abundant RA response element, the RA receptor beta D5 response element [42]. Sequences for the DR5 probe were (forward) AGGGTTCACCGAAAGTTCACTCG and (reverse) CGAGTGAACTTTCGGTGAACCCT. The negative control consisted of substituting an equal volume of extraction buffer for the nuclear extract.

### Statistics

Data are reported as mean ± S.E.M. if normally distributed and median with interquartile ranges (25% - 75%) if not normally distributed. Expression differences between groups were analyzed using Student’s *t*-test for normally distributed data. Data that were not normally distributed were analyzed using non-parametric statistical tests (Mann–Whitney). Differences were accepted as statistically significant when P < 0.05.

## Results

### Murine model of gestational tobacco toxin exposure

We adapted a published [43-45] adult murine model of cigarette smoke exposure to examine the effects of prenatal tobacco toxin exposure in juvenile mice. Total particulates in this model were 310.6 mg/mm^3^. Cigarette smoke-exposed pregnant adult female mice (N = 8) had significantly (P < 0.05) greater plasma levels of the nicotine metabolite cotinine compared to tobacco-naïve mice (N = 8): the median cotinine plasma level in the smoke-exposed mice was 30 ng/ml (interquartile range 8.9 ng/ml – 45.4 ng/ml) compared to 3.0 ng/ml (interquartile range 2.6 ng/ml – 3.3 ng/ml) in the nonsmoking mice. Similarly, median placenta cotinine levels were also significantly (P < 0.05) increased in the smoke-exposed mice: 6.7 ng/ml (interquartile range 2.9 ng/ml – 10.1 ng/ml), whereas no placental cotinine was detected in the tobacco-naïve mice. The average litter size of the smoke-exposed females was 6.5 (± 0.38) pups, which was significantly (P = 0.028) smaller than the average litter size of the nonsmoker mice: 7.8 (± 0.39) pups.

**Figure 1:**
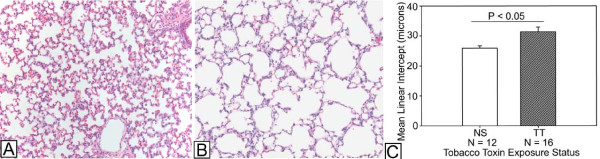
**Increased airspace size in mice with tobacco toxin (TT) exposure during lung development compared to tobacco-naïve (NS) mice.A.** Representative section of lung tissue stained with hematoxylin and eosin (H&E) from postnatal day 14 (P14) NS mouse (Magnification, 20X) showing normal alveolar size. **B.** H&E stain of representative section of lung tissue from P14 TT mouse (Magnification, 20X) showing enlarged airspace size, consistent with alveolarization defect. **C.** The mean linear intercept, which reflects average distance between gas exchange elements, is significantly larger in TT mice compared to NS mice. Data comparison performed using Student’s *t*-test. Abbreviation: N = number of mice in group. Data are mean ± SEM.

In our model, exposure to maternal smoking caused significantly (P < 0.001) decreased weights in the offspring that persisted until at least postnatal day 5 (P5), with average body weight 3.3 gm (± 0.09 gm) in the non-exposed pups and 2.5 gm (± 0.08 gm) in the mice with prenatal tobacco toxin exposure. Additionally, our model showed that tobacco toxin exposure during development significantly (P = 0.009) increased mean linear intercept (MLI) at P14, consistent with increased average distance between gas exchange elements in the distal lung, in these mice compared to age-matched tobacco-naïve mice (Figure 1).

### Maternal smoking decreases expression of components of the retinoic acid signaling pathway in a murine model

We tested the hypothesis that maternal smoking causes abnormalities in the RA signaling pathway in the lungs of offspring by first using quantitative PCR to compare mRNA expression of RA signaling pathway components in distal lung tissue RNA extracted from juvenile mice (postnatal ages P0 – P10) with/without tobacco toxin exposure during development We used 18 S ribosomal RNA expression as an endogenous control since it is not regulated during lung development.

**Figure 2:**
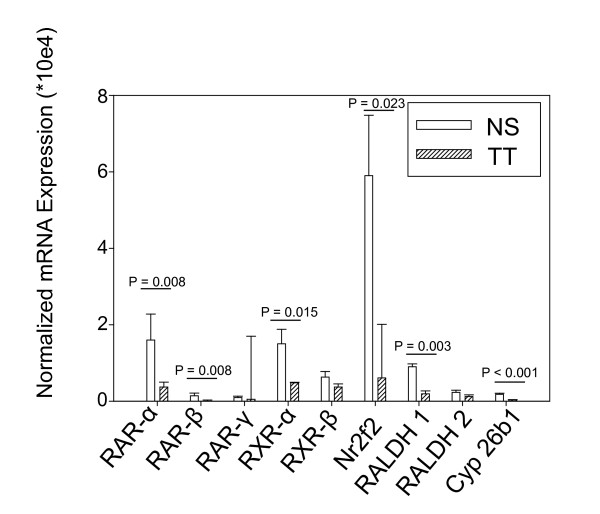
**Quantitative PCR analysis of RA pathway components at postnatal day 5 (P5) comparing mice with tobacco toxin exposure during lung development (TT, N = 9) to tobacco naïve (NS, N = 5) mice.** All results normalized to expression of 18 S. Compared to NS mice, lung tissue samples from mice with TT had decreased expression of retinoic acid receptors (RAR) alpha and beta, retinoid X receptor (RXR) alpha, nuclear receptor family 2, group F, member 2 (Nr2f2) – also known as chicken ovalbumin upstream promoter transcription factor 2 (COUP TF 2), retinaldehyde dehydrogenase 1 (Raldh1), and cytochrome P450 26b1 (Cyp 26b1). Data are presented as mean ± SEM. NS = tobacco naïve. Data comparison performed using Student’s *t*-test for normally distributed data and Mann–Whitney for non-normally distributed data.

This analysis showed that mice with exposure to tobacco toxins during lung development had significantly decreased mRNA for multiple components of the RA signaling pathway at P5. These included RA receptor alpha (Rara, P = 0.008), RA receptor beta (Rarb, P = 0.008) retinoid-X receptor alpha (Rxra, P = 0.015), retinaldehyde dehydrogenase-1 (Raldh1, P = 0.003), cytochrome P450 26b1 (P < 0.001), and nuclear receptor family 2, group F, member 2 (Nr2f2) – also known as chicken ovalbumin upstream transcription factor 2 (COUP TF2, P = 0.023) expression on day P5 compared to tobacco naïve mice (Figure 2). Additionally, mice with tobacco toxin exposure also had decreased mRNA expression for retinoid X receptor gamma at P7 (Rxrg, P = 0.021). Details of the mRNA expression analyses are given in Supplemental Data ( Additional file 1: Table S1).

**Figure 3:**
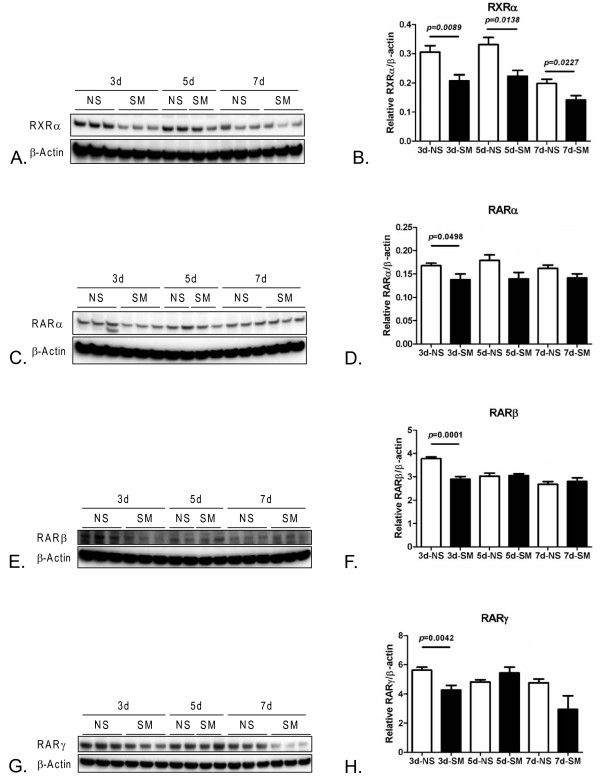
**Western blot analyses for retinoid X receptor alpha (RXRalpha), retinoic acid receptor alpha (RARalpha), retinoic acid receptor beta (RARbeta), and retinoic acid receptor gamma (RARgamma) expression in distal lung tissue from mice with and without tobacco toxin exposure during development (TT) at postnatal age P3, P5, and P7.** Representative Western blots for RXRalpha **(A)**, RARalpha **(C)**, RARbeta **(E)**, and RARgamma **(G)**. Panels **B.,D,F,** and **H:** Densitometry analyses of Western blots on distal lung tissue from juvenile mice at postnatal ages P3 with (N = 6) and without (N = 6) TT, P5 (N = 4 each with/without TT), and P7 with (N = 6) and without (N = 5) TT. Compared to tobacco-naïve mice, TT pups expressed significantly decreased RXRalpha at each time point (B): at P3, P = 0.0089, at P5 P = 0.0138, and at P7 P = 0.0227. Decreased expression in the TT mice was also seen for RARalpha, although differences between groups were less than those for RXRalpha, with borderline significance observed at P3 (P = 0.0498) and trends for decreased expression at P5 (P = 0.0709) at P5 and P7 (P = 0.0866) (D). In contrast, TT mice only showed significantly decreased RARbeta at P3 (P < 0.001) (F). The expression of RARgamma was similar to that of RARbeta, with significant decreases seen in the TT mice at P3 (P = 0.0042), with a trend toward decrease at P7 (P = 0.0728). Data comparison performed using Student’s *t*-test. Abbreviations: RXR = retinoid X receptor, RAR = retinoic acid receptor.

Since tobacco toxin exposure during development caused abnormal mRNA expression for several of the elements of the RA signaling pathway, we determined if protein expression was also affected by tobacco toxin exposure. Western blot analysis was performed for retinoid X receptor alpha (RXRalpha), RA receptor alpha (RARalpha), RA receptor beta (RARbeta), and RA receptor gamma (RARgamma) in protein extracted from distal lung tissue from mice with and without tobacco toxin exposure during development (N ≥ 4 each group) at time points P3, P5, and P7. Similar to the mRNA analysis, these studies showed significantly decreased protein expression for components of the RA signaling pathway in mice with tobacco toxin exposure (Figure 3). However, the kinetics of the decreased protein expression differed from that of the mRNA expression. At P3, RXRalpha (P = 0.0089), RARbeta (P = 0.0001), RARgamma (P = 0.0042), and RARalpha (P = 0.0498) were all significantly decreased in the mice with tobacco toxin exposure. In contrast to the mRNA expression pattern, only RXRalpha was significantly decreased (P = 0.0138) at P5. At P7, RXRalpha was significantly decreased (P = 0.0227), with a trend toward decreased RARgamma (P = 0.0728) in the tobacco toxin exposed mice. Thus, both the mRNA and protein analysis showed that tobacco toxin exposure during development caused significantly decreased expression of multiple components of the RA signaling system in the postnatal murine lung.

### Cigarette smoke condensate decreases RA response element activation *in vitro*

**Figure 4:**
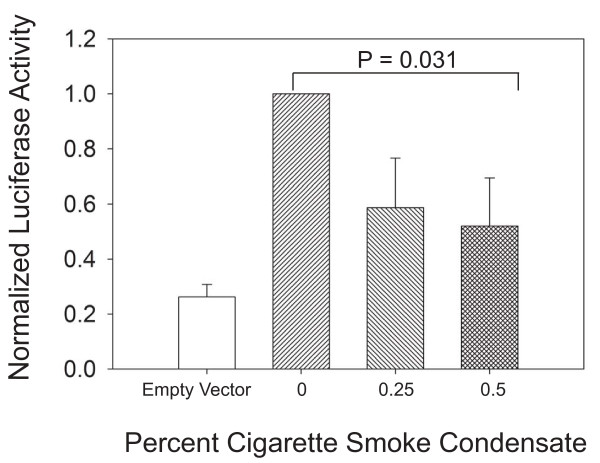
**Cigarette smoke condensate (CSC) significantly (P < 0.05) decreases retinoic acid-stimulated (1 μM all groups) response element (RARE) activity as detected by a luciferase reporter plasmid in A549 cells.** Luciferase activity was normalized to that of the retinoic acid-stimulated cultures without CSC. Data (mean ± SEM) are from 4 independent experiments. Data comparison performed using Student’s *t*-test.

RA signaling requires the formation of a RAR-RXR heterodimer, which then binds to a RA response element in RA-regulated genes to modify transcription. Heterodimer binding is down-regulated by Nr2f1 and Nr2f2, which compete for the RXRs. Exposure to tobacco toxins decreased expression for multiple RA pathway components, including RARs, RXRs, and Nr2f2. The qPCR and Western blot analyses indicated that the RA system is affected by tobacco toxin exposure, but did not determine whether such exposures are functionally significant. We therefore next examined whether lipid-soluble components of cigarette smoke could possibly affect the activation of a RA response element in an *in vitro* cell culture system, using the pulmonary type II cell-like cell line A549. We examined RA response element activation by transfecting these cultures with a commercially available luciferase reporter plasmid containing the RA response element. This plasmid showed robust luciferase activity following stimulation with 1 μM RA, with minimal activity demonstrated after transfection with the plasmid backbone (Figure 4). This luciferase activity was significantly (P < 0.05) decreased in a dose-dependent fashion by treating the cultures with lipid-soluble cigarette smoke condensate (CSC, Figure 4). Toxicity analysis by LDH release showed no significant LDH release at the 0, 0.25, and 0.50% CSC. This analysis did show a significant increase in LDH at 0.75% CSC, which was a greater concentration than that which significantly decreased luciferase activity in the A549 cells (data not shown). Therefore, the decreased luciferase activity was not explained by cell toxicity alone.

**Figure 5:**
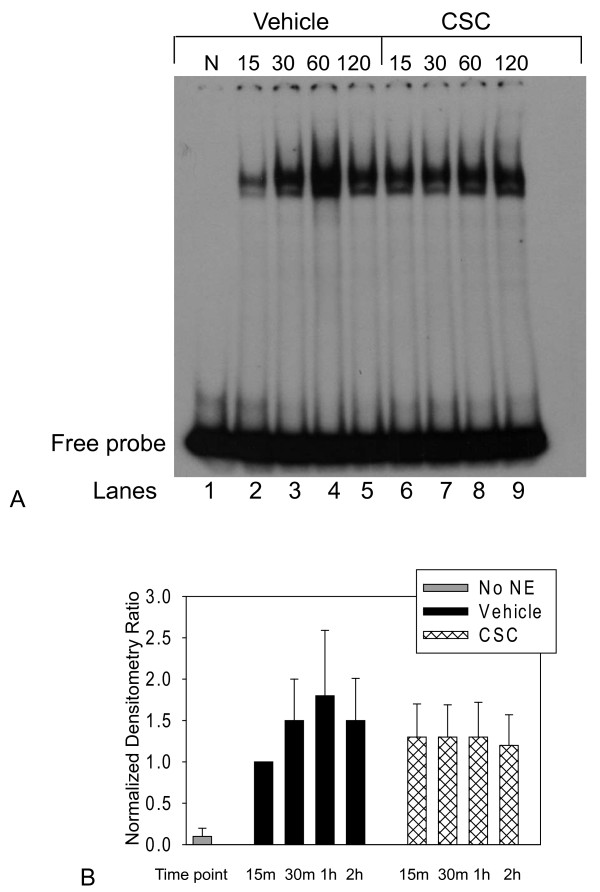
**Cigarette smoke condensate (CSC) decreases retinoic acid response element binding*in vitro.*A:** Representative electrophoretic mobility shift assay (EMSA) showing decreased binding of nuclear extracts from A549 cells to the DR5 retinoic acid response element after 1 μM retinoic acid and vehicle (lanes 2 – 5) or 0.50% cigarette smoke condensate (CSC, lanes 6–9) treatment followed by second CSC dose of 0.50% 14 h after the first, with extracts harvested at 15 – 120 min. following this second dose. Similar results were also shown in an independent replication. **B:** Relative densities of A549 cell nuclear extract/DR5 retinoic acid response element complexes (normalized to free probe) following stimulation with 1 μM retinoic acid and either vehicle or 0.5% CSC as outlined for panel A. Data are mean ± SEM from two independent experiments. Abbreviations: N = negative control lane (lane 1) with equivolume extraction buffer substituted for the nuclear extract; numbers indicate time course in minutes, lane numbers for samples indicated below figure, NE = nuclear extract, CSC = cigarette smoke condensate.

Decreased RA response element activation could be caused by decreased binding of the RAR-RXR heterodimer to the RA response element, or by decreased functioning of the normally bound RAR-RXR heterodimer. We tested whether decreased RAR-RXR binding contributed to the decreased RA response activation observed in our CSC-treated cell cultures by performing electrophoretic mobility shift assays (EMSA) to determine whether CSC could decrease binding of nuclear extracts from A549 cells to the well-characterized consensus binding sequence of the D5 RA response element, which is the most common of the RA response elements [42]. EMSA confirmed that nuclear extract from RA-treated (1 μM) A549 cells exhibited binding to the RA response element, as evidenced by a single band in the vehicle-treated groups (lanes 2 – 5) that was not present without addition of nuclear extract (lane 1). Nuclear protein binding was visibly decreased following treatment with CSC 0.50% at 30 (lane 7) and 60 (lane 8) minutes compared with vehicle control at these same time points (lanes 3 and 4, Figure 5A). Quantification of binding in two different EMSAs supported a decrease in binding at 30, 60, and 120 minutes after CSC treatment compared with vehicle control (Figure 5B). Thus, EMSA indicated that decreased nuclear protein binding to the RA response elements might contribute to the decreased activity of the RA response element following CSC.

### Maternal smoking decreases expression of RA-regulated genes *in vivo*

**Figure 6:**
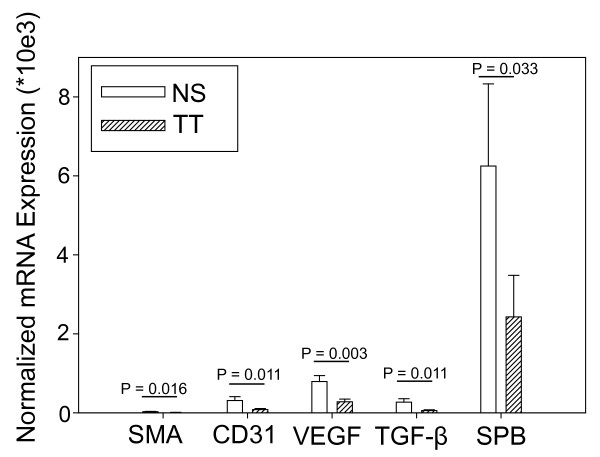
**Real-time PCR analysis of postnatal day 5 murine lung tissue showing significantly decreased expression of mRNA (normalized for 18 S expression) for genes regulated by retinoic acid that modulate alveolarization and/or postnatal lung function in mice exposed to tobacco toxin exposure during lung development (TT, N = 5) compared to tobacco-naïve mice (NS, N = 9).** Abbreviations: SMA = smooth muscle actin, CD31 = platelet endothelial cell adhesion molecule, VEGF = vascular endothelial cell growth factor alpha, TGF-β = transforming growth factor beta, SPB = surfactant apoprotein beta. Data are presented as mean ± SEM. Data comparison performed with Student’s *t*-test for normally distributed data and Mann–Whitney for non-normally distributed data.

Since our *in vitro* studies suggested that tobacco toxins could decrease the activity of the RA signaling pathway, we next used our murine model to determine whether there was evidence for decreased RA signaling in mice with tobacco toxin exposure during lung development. RA signaling modulates several aspects of lung maturation and postnatal growth including alveolarization and surfactant apoprotein B expression. We focused these studies on the P5 time point since that had previously shown the greatest number of abnormalities in RA pathway component expression in the mice with tobacco toxin exposure. We used qPCR to compare the expression of mRNA for a panel of five genes that are regulated by RA [46]: smooth muscle actin (SMA), transforming growth factor-β (TGFβ), platelet endothelial cell adhesion molecule (PECAM, CD31), vascular endothelial growth factor (VEGF), and surfactant apoprotein B (SPB). This analysis (Figure 6) showed that mice exposed to tobacco toxins during lung development had significantly decreased mRNA expression for SMA (P = 0.016), TGF-β (P = 0.011), PECAM (P = 0.011), VEGF (P = 0.003), and SPB (P = 0.033) at P5. Details of the mRNA expression analyses are given in Supplemental Data ( Additional file 2: Table S2). Thus, the qPCR analyses indicate that exposure to tobacco toxins during lung development via maternal smoking could decrease the postnatal functioning of the RA pathway in our murine model.

## Discussion

This study shows that exposure to tobacco toxins via maternal smoking during lung development causes postnatal abnormalities in the RA signaling system. Our model of murine cigarette smoke exposure during pregnancy caused significantly increased levels of a nicotine metabolite, cotinine, in both the maternal serum and placental tissues. Of note, our analysis also detected a low-level of cotinine in the serum, but not the placental tissues, of the nonsmoking adult mice. The concentration of serum cotinine in these animals was very low, near the limits of detection of our assay, and corresponded to similar findings reported in other rodent models [47,48]. The source of cotinine in the nonsmoking mice is not clear, however, it is possible that it may be a dietary source and/or vitamin supplement – niacin has been reported to cross-react with cotinine assays, and low levels of nicotine have been demonstrated in some none-tobacco plants [49,50]. Similar to what other investigators have reported [51], mice with tobacco toxin exposure during development had decreased weights compared to tobacco-naïve pups. In our model, the differences in weights were detected through postnatal day 5 (P5).

Regarding the histological changes in the juvenile mice with developmental tobacco toxin exposure, our model caused a significant increase in the mean linear intercept, which is an estimate of the average distance between gas exchange elements, at P14. This is consistent with defective alveolarization in the mice with tobacco toxin exposure during lung development. While commonly used to compare distal lung architecture [43,45,52-64], the mean linear intercept does not provide an accurate assessment of the irregularly shaped alveolus [34]. Additionally, it is not as sensitive as other morphometric approaches, especially if the changes in tissue architecture are not uniformly distributed throughout the lung parenchyma [34,65]. Thus, our analysis may have underestimated the effects of tobacco toxin exposure on the immature lung architecture. Of note, cigarette smoke exposure in adult mice frequently requires several months to establish emphysematous-like changes [43-45,62,63]. Our finding of defective alveolarization following tobacco toxin exposure during gestation and neonatal lung development, similar to that reported by other investigators [57,66], suggests that the immature lung may have heightened susceptibility to the effects of tobacco toxins.

The histological abnormalities were associated with decreased mRNA and protein expression of several RA signaling pathway elements in distal lung tissue from mice with tobacco toxin exposure during lung development. This analysis examined time points which encompassed the start of rapid alveolarization in the mouse, of which retinoids are critical mediators [18,21,22,67,68]. Unexpectedly, these abnormalities were not greatest immediately after birth, but instead were greatest between P3 to P5, and diminished thereafter. Thus, the effects of maternal smoking persisted in the juvenile mice for several days after birth. The significance of the P5 time point is that this is immediately before the onset of rapid alveolarization in the mouse [69]. Therefore, abnormalities at this time point might have a disproportionately greater impact on alveolarization, such as those shown in our histological analyses. Our data suggest that the effects of developmental exposure to tobacco toxins are greatest at this important postnatal period of lung maturation. The onset of alveolarization is characterized by down-regulation of Rara, Rarb, and Rarg isoforms in the lung [70,71]. However, the roles of the retinoids in alveolarization are complex – absence of Rara and/or Rarg causes defective alveolarization [22,72]. These studies indicate that, normal alveolarization requires precise regulation of the kinetics and abundance of retinoids [21,71,73]. Our data suggest that tobacco toxin exposure during development disrupts this highly regulated process.

We tested whether the decrease in RA pathway components could possibly be functionally significant by examining an *in vitro* cell culture model. Our analysis of the expression of RA pathway components showed that a negative regulator of RAR signaling, Nr2f2, was decreased in the mice with tobacco toxin exposure. The decrease in a negative regulator would be expected to increase RA signaling. However, the decreased expression of the other components of the RA pathway would tend to decrease RA signaling. Thus, our RNA expression findings did not clearly indicate whether RA signaling would be affected by cigarette smoke exposure. We therefore determined whether components of cigarette smoke could possibly affect activation of a RA response element in an *in vitro* cell culture model. Treatment of the type II-like cell line, A549, with cigarette smoke condensate (CSC) decreased activation of a RA response element in a luciferase reporter plasmid, and EMSA demonstrated that CSC decreased binding of nuclear extracts prepared from A549 cells to the most common RA response element, the DR5. Such decreased binding and RA response element activation would tend to reduce RA-regulated gene transcription. Therefore, our cell culture data suggested that it would be feasible for tobacco toxin exposure to affect the functioning of the RA signaling pathway *in vivo*.

Since our *in vitro* model suggested that components of cigarette smoke might decrease RA signaling in lung cells, we examined our *in vivo* model of murine developmental tobacco toxin exposure for similar evidence of decreased RA pathway function by examining mRNA expression of RA-regulated genes in lung tissue from mice with developmental tobacco toxin exposure. This analysis showed that prenatal cigarette smoke exposure decreased mRNA for SMA [74,75], CD31 [76], TGF-β [77,78], VEGF [79], and SPB [80,81] – all of which modulate postnatal alveolarization and/or lung function. Additionally, RA modulates the expression of all of these genes [46]. The effects of RA on these genes can vary with the specific model studied. RA modulation of TGF-β provides a good example of the context-dependent effects of RA, since RA has been shown to both decrease [82,83] and increase [84-86] TGF-β expression. RA can increase transcription either by directly activating RA response elements in the upstream promoter region, as observed with SPB [23], and/or by modulating the activity of transcription factors such as hypoxia-inducible factor-1α [87]or Sp1 [88,89], as is the case with VEGF, SMA [90], and PECAM [91,92]. The two mechanisms are not mutually exclusive. For example, RA-modulation of SPB includes activation of a promoter RA response element [23] and activation of transcription factors TTF-1 [93,94], signal transducers and activators of transcription-3 (STAT3), and Janus family tyrosine kinase-1 (JAK1) [95]. Additionally, the effects of RA can be amplified by RA-mediated increase in growth factor receptors, which occurs with VEGF [96] and TGF-β. Thus, our *in vitro* and *in vivo* data support the hypothesis that components of cigarette smoke can decrease the activity of the RA signaling pathway in the immature lung.

The retinoids are critical mediators of lung development and maturation [68,70,97-103], including alveolarization [20,22,72]. However, this signaling pathway has not been extensively studied in relation to cigarette smoking. Animal models have shown decreased serum vitamin A levels following cigarette smoke exposure in adult rats. The serum vitamin A levels were inversely correlated with the amount of emphysematous changes in lung tissue [26]. Moreover, a recent study in adult C57Bl/6 mice showed that vitamin A deficiency significantly increased the degree of cigarette smoke-induced lung damage in comparison to cigarette smoke exposure in vitamin A-replete mice [104]. In humans, several studies have reported that cigarette smokers have decreased serum beta carotene [27,28]; one large study has also shown decreased serum retinol levels in current smokers compared to former smokers [28]. Cigarette smoking increases methylation of the promoter of the gene encoding RAR-beta, thereby decreasing expression of this receptor [105-107]. Additionally, squamous metaplasia is observed in both vitamin A deficiency and smokers [103], and a clinical trial of supplementing 13-cis RA showed improved lung epithelial histology [108]. However, clinical trials of supplementing beta carotene in active smokers showed a trend toward increased lung cancer in patients receiving beta carotene. Subsequent analysis suggests that the mechanism of this increase could be due to down-regulation of RA signaling caused by excess beta carotene [109,110].

Our data extend the findings of other investigators studying the effects of maternal smoking in animal models. Both maternal nicotine treatment and cigarette smoke exposure disrupt alveolarization in rats, guinea pigs, mice, and rhesus monkeys [66,111-115]. Of note, a recent report by Singh and colleagues demonstrated that maternal smoking decreased cAMP in the lung tissue of the offspring [66]. Although retinoids were not specifically examined in that study, cAMP can modulate the activity of Rara through protein kinase A sites in the *Rara* promoter [116-118].

Our murine model has several limitations. First, the control mice were exposed to filtered air only, and not put into unused smoking chambers (sham-exposed). Filtered air as the control exposure has been used by other investigators examining cigarette smoke exposure in adult mice, and has been found to be equivalent to sham exposures [44,45,62]. However, we cannot rule out a possible contribution from the enclosure in the smoking chamber in our tobacco toxin-exposed mice. Additionally, the maternal cigarette exposure was continued following parturition. This was necessary because our preliminary experiments established that maternal behavior was very poor if the cigarette exposure was abruptly discontinued following delivery. Thus, our model did not isolate the tobacco toxin exposure to gestation. Since lung development in the mouse continues for at least two weeks after birth [69,119], our findings remain informative regarding the effects of tobacco toxin exposure in the developing lung despite this limitation. Another limitation is that our *in vitro* cell culture experiments provide supportive, but not conclusive, evidence of decreased RA signaling following tobacco toxin exposure during lung development. Definitive experiments to prove that tobacco toxin exposure decreases RA signaling in the immature lung would require a detailed analysis of *in vivo* signaling pathway activity, and are beyond the scope of this report.

## Conclusions

Our data show that exposure to tobacco toxins during development decreases the expression of RA pathway elements in the immature lung, which indicates that this important signaling pathway is vulnerable to the effects of maternal smoking during pregnancy. The decreases were most pronounced immediately before the onset of rapid alveolarization, at post-natal days 3–5. *In vitro* experiments using a luciferase reporter plasmid showed that CSC decreased activation of the RA response element. Additionally, tobacco toxin exposure during development decreased the expression of RA-regulated genes, suggestive of decreased RA pathway functioning in the smoke-exposed mice. We therefore propose that decreased RA pathway activity may be a mechanism of lung injury caused by prenatal tobacco toxin exposure, and may represent a therapeutic target for pediatric lung disease that arises from maternal smoke exposure.

## Competing interests

None of the authors have competing interests.

## Authors’ contributions

SEM, LAS, and YP assisted in experimental design, performed experiments, and assisted in the writing of the manuscript. CV, CHA and RMB performed experiments and assisted in the writing of the manuscript. WC assisted in experimental design and the writing of the manuscript. KJH developed and coordinated the experimental design and assisted in writing the manuscript. All authors read and approved the final manuscript.

## Authors’ information

WC is a senior member of the research faculty of Boston University Medical Center in Boston, MA. He is an expert on the roles of retinoic acid in lung development. CAV is a senior researcher in pharmacology at Children’s Mercy Hospital and Clinics in Kansas City, MO. RMB and KJH are physician-researchers at the Brigham and Women’s Hospital in Boston, MA.

## Supplementary Material

Additional file 1 **Table S1.** Tobacco toxin exposure during development causes abnormal postnatal retinoic acid receptor expression. Click here for file

Additional file 2 **Table S2.** Prenatal tobacco toxin exposure causes abnormal postnatal expression of genes modulated by retinoic acid. Click here for file

## Author details

^1^Department of Medicine, Division of Pulmonary and Critical Care, Brigham and Women’ s Hospital, Boston, MA, USA. ^2^Pulmonary Center of Boston University School of Medicine, Boston, MA, USA. ^3^Division of Pediatric Clinical Pharmacology, Children’ s Mercy Hospital and Clinics, Kansas City, MO, USA.
