# Supplementary material for: Maternal smoking and the retinoid pathway in the developing lung
Source: Respir Res. 2012 Jun 1;13(1):42. doi: 10.1186/1465-9921-13-42 (PMC3479035; doi:10.1186/1465-9921-13-42)
Supplement: Additional file 1 — Table S1. Tobacco toxin exposure during development causes abnormal postnatal retinoic acid receptor expression. [file 1465-9921-13-42-S1.doc]

Supplemental Data Table 1: Tobacco toxin exposure during development causes abnormal postnatal retinoic acid receptor expression

| Pathway  component | Day of birth (P0) | | Postnatal day 3 | | Postnatal day 5 | | Postnatal day 7 | | Postnatal day 10 | |
| --- | --- | --- | --- | --- | --- | --- | --- | --- | --- | --- |
|  | NS = 7 | TT = 9 | NS = 8 | TT = 7 - 8 | NS = 5 | TT = 8 - 9 | NS = 7 - 10 | TT = 9 - 10 | NS = 9 - 10 | TT = 8 |
| Rara | 9.9e-05  (6.8e-05 -  6.4e-05 | 1.1e-04  (6.4e-05 –  2.5e-04) | 1.4e-04  (2.8e-05) | 1.3e-04  (2.9e-05) | 1.6e-04 (8.1e-05 – 2.6e-04) | 3.7e-05*  (1.6e-05 –  5.9e-05)  P = 0.008 | 7.0e-05  (2.4e-05) | 8.4e-05  (1.4e-05) | 1.1e-04  (7.4e-05 –  1.4e-05) | 9.1e-05  (6.4e-05 –  1.0e-04) |
| Rarb | 9.0e-06  (1.7e-06) | 6.2e-06  (1.4e-06) | 3.0e-06  (2.3e-06 –  7.1e-06) | 2.5e-06  (1.4e-06 –  5.9e-06) | 1.4e-05  (1.3e-05 –  2.3e-05) | 9.6e-07*  (7.9e-07 –  1.1e-05)  P = 0.008 | 3.4e-06  (1.2 e-06) | 4.3e-06  (9.3e-07) | 7.3e-06  (1.8e-06) | 7.3e-06  (1.6e-06) |
| Rarg | 3.0e-06  (2.4e-08 –  1.7e-05) | 6.7e-06  (5.7e-06 –  1.7e-05) | 5.3e-06  (1.3e-06) | 9.2e-06  (2.9e-06) | 9.6e-06  (3.6e-06) | 4.9e-06  (1.7e-06) | 1.1e-05  (6.5e-06 –  3.2e-05) | 9.8e-06  (6.5e-06 –  2.8e-05) | 8.3e-06  (4.1e-06 –  1.2e-05) | 8.6 e-06  (4.3e-06 – 9.3e-06) |
| Rxra | 3.4e-05  (6.7e-06) | 2.9e-05  (5.2e-06) | 1.0e-04  (7.7e-05 –  1.4e-04) | 7.3e-05  (6.2e-05 –  8.8e-05) | 1.5e-04  (3.8e-05) | 4.8e-05*  (1.6e-06)  P = 0.015 | 5.8e-05  (1.5e-05) | 9.6e-05  (1.6e-05) | 1.2e-04  (2.0e-05) | 7.9e-05  (1.3e-05) |
| Rxrb | 4.8e-05  (9.0e-06) | 6.1e-05  (1.3e-05) | 6.5e-05  (1.4e-05) | 5.4e-05  (8.6e-06) | 6.3e-05  (1.5e-05) | 3.7e-05  (8.1e-06) | 8.1e-05  (6.5e-05 –  1.2e-04) | 1.6e-04  (7.9e-5 – 2.5e-04) | 2.0e-05  (3.4 e-06) | 9.0e-06  (3.2e-06) |
| Rxrg | 3.9e-8  (2.1e-8 –  1.7e-08) | 2.0e-08  (1.7e-08 –  5.0e-08) | 3.7e-07  (2.4e-07 –  1.1e-06) | 4.1e-07  (2.3e-07 –  7.0e-07) | 1.7e-06  (2.6e-07) | 9.3e-07  (2.7e-07) | 2.9e-07  (4.2e-08) | 1.6e-08  (3.0e-08) *  P = 0.021 | 3.2e—07  (8.5e-08) | 2.6e-7  (6.5e-08) |
| Raldh1 | 1.1e-04  (1.2e-05 –  1.3e-04) | 5.4e-05  (3.4e-05 –  6.4 e-05) | 1.5e-05  (3.6e-06)) | 7.7e-06  (2.9e-06) | 9.0e-05  (7.0e-05 –  1.0e-04) | 1.9e-05  (1.4 e-05 –  6.0e-05)  P = 0 003 | 2.6e-06  (2.5e-06 –  6.4e-06) | 3.0e-06  (2.1e-06 –  4.1e-06) | 5.5 e-05  (3.4 e-05 –  6.1e-05) | 5.1 e-05  (2.0e-05 –  7.1 e-05) |

Table provides relative amounts of mRNA assessed by quantitative PCR, normalized to 18S expression. Values are mean (± SEM) if normally distributed and median (interquartile range) if not normally distributed. Normally distributed data were compared using Student’s t-test, and not normally distributed data were compared using Mann-Whitney. Abbreviations: NS designates tobacco-naïve mice, TT designates mice with developmental exposure to tobacco toxins, Rara designates retinoic acid (RA) receptor alpha, Rarb designates RA receptor beta, Rarg designates RA receptor gamma, Rxra designates retinoid X receptor alpha, Rxrb designates retinoid X receptor beta, Rxrg designates retinoid X receptor gamma, and Raldh1 designates retinaldehyde dehydrogenase-1. P values provided under TT column for comparisons showing significant difference (P < 0.05) between TT and NS.
