# Supplementary material for: Maternal smoking and the retinoid pathway in the developing lung
Source: Respir Res. 2012 Jun 1;13(1):42. doi: 10.1186/1465-9921-13-42 (PMC3479035; doi:10.1186/1465-9921-13-42)
Supplement: Additional file 2 — Table S2. Prenatal tobacco toxin exposure causes abnormal postnatal expression of genes modulated by retinoic acid. [file 1465-9921-13-42-S2.doc]

| RA-modulated  gene | Tobacco-naïve  N = 5 | Tobacco Toxin Exposed  N = 9 | P value |
| --- | --- | --- | --- |
| SMA | 2.2e-05 (1.9e-05 – 3.4e-05) | 2.2e-06 (1.6e-06 – 1.0e-05) | 0.016 |
| CD31 | 2.1e-04 (1.8e-04 – 4.0e-04) | 3.1e-05 (2.0e-05 – 1.2e-04) | 0.011 |
| VEGF | 7.9e-04 (1.5e-04) | 2.8e-04 (6.7e-05) | 0.003 |
| TGF-β | 1.8e-04 (1.6e-04 – 3.6e-04) | 4.4e-05 (2.8e-05 – 1.1e-04) | 0.011 |
| SPB | 4.8e-03 (2.9e-03 – 8.6e-03) | 1.3e-03 (4.8e-04 – 2.8e-03) | 0.033 |

Supplemental Data Table 2: Prenatal tobacco toxin exposure causes abnormal postnatal

expression of genes modulated by retinoic acid

Table provides relative amounts of mRNA assessed by quantitative PCR, normalized to 18S expression, in distal lung samples from mice, aged postnatal day 5, with/without tobacco toxin exposure during development. Values are mean (± SEM) if normally distributed and median (interquartile range) if not normally distributed. Normally distributed data were compared using Student’s t-test, and not normally distributed data were compared using Mann-Whitney. Abbreviations: SMA designates smooth muscle actin, CD31 designates cluster of differentiation 31 (also called platelet endothelial cell adhesion molecule), VEGF designates vascular endothelial growth factor, TGF-β designates transforming growth factor-beta, and SPB designates surfactant apoprotein B. P values are provided for comparison of expression levels between the tobacco naïve and tobacco toxin-exposed mice.
